# Assessing Household Economic Vulnerability in HIV-Affected Communities in Five Regions of Côte d'Ivoire

**DOI:** 10.1371/journal.pone.0163285

**Published:** 2016-09-21

**Authors:** Holly M. Burke, Whitney Moret, Samuel Field, Mario Chen, Yanwu Zeng, Firmin M. Seka

**Affiliations:** 1 Global Health, Population and Nutrition Department, Durham, North Carolina, United States of America; 2 Social and Economic Development Department, Washington, D.C., United States of America; 3 Synergie Expertise, Abidjan, Côte d’Ivoire; Tulane University School of Public Health and Tropical Medicine, UNITED STATES

## Abstract

The objective of this study was to identify and describe levels of household economic vulnerability in HIV-affected communities in Côte d’Ivoire, defined as those with a high prevalence of HIV and large numbers of orphans and vulnerable children. We conducted a cross-sectional survey of 3,749 households in five health regions of Côte d’Ivoire. Using principal component analysis, we attempted to identify sets of correlated vulnerabilities and derive a small number of composite scores to create an index for targeting interventions to vulnerable populations. The 65 vulnerability measures examined did not cluster in ways that would allow for the creation of a small number of composite measures. Instead, we found that households face numerous unique pathways to vulnerability.

## Introduction

Among West African countries, Côte d’Ivoire has historically been the hardest hit by HIV. With a current estimated prevalence of 2.7 per cent, Côte d’Ivoire is home to approximately 400,000 children orphaned by AIDS [1]. Although Côte d’Ivoire has made laudable progress in the fight against HIV, the majority of the population remains economically vulnerable, leaving those affected by HIV less able to cope with its health and economic consequences. In response, the United States Agency for International Development (USAID) and President's Emergency Plan for AIDS Relief (PEPFAR) are working with local implementing agencies and the Côte d’Ivoire Ministry of Health to engage households with members infected with or affected by HIV in economic strengthening (ES) activities. A key component in PEPFAR’s programming for orphans and vulnerable children (OVC), ES has been identified as a structural intervention to address the upstream effects of poverty on HIV vulnerability as well as a means for mitigating HIV-related outcomes. ES interventions include financial education, formal and informal microfinance, vocational training, and others designed to reduce the economic vulnerability of households and improve their resilience to HIV and AIDS as well as economic shocks more broadly.

**Fig 1:**
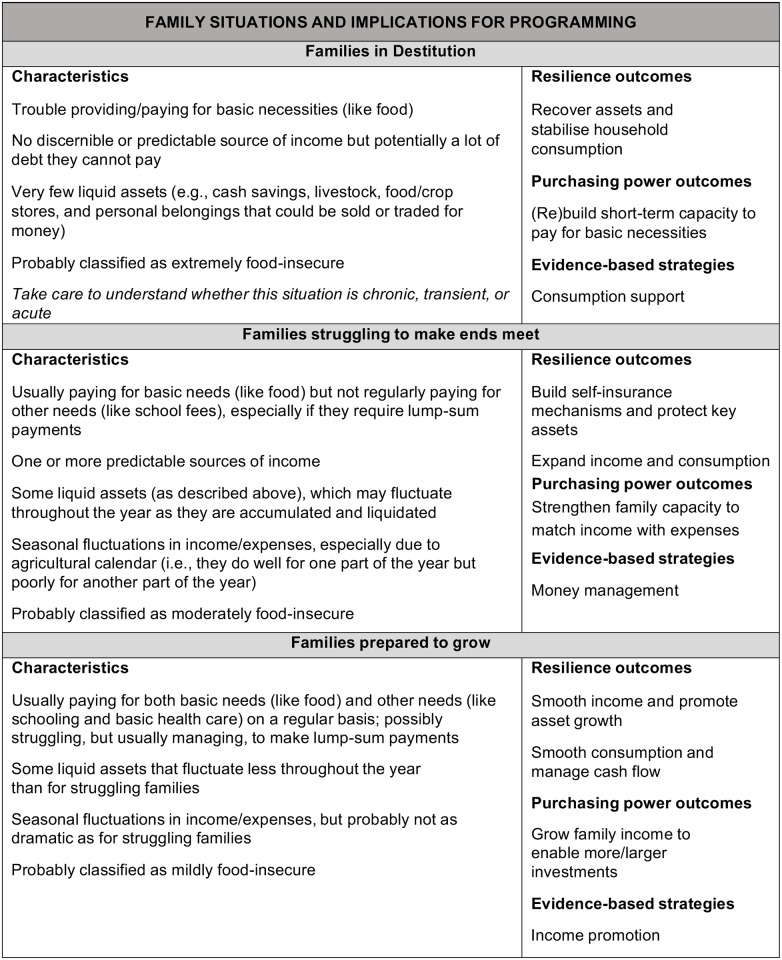
PEPFAR economic strengthening categories (see p. 47 in [2]).

PEPFAR guidance on ES programming recommends a graduated approach where interventions are targeted and sequenced to promote rising socioeconomic status [2]. This guidance is based on existing research about how households manage their asset portfolios in response to different kinds of shocks and stresses, including HIV [3, 4]. It places households into three status categories: destitute, struggling to make ends meet, and ready to grow (Fig 1). The USAID/PEPFAR Health Office commissioned this study to more precisely target its programming to beneficiaries according to the PEPFAR ES categories. The results from this research will help shape the development and provision of ES services for vulnerable households in Côte d’Ivoire.

Economic status, as measured by poverty level, is a static metric. The concept of vulnerability offers a more forward-looking means to assess populations by measuring how risks and shocks can affect future status [5]. An understanding of vulnerability helps to inform program design and targeting. Our study used a sustainable livelihoods approach to vulnerability assessment to get a snapshot of several domains of economic vulnerability beyond poverty statistics. The literature on sustainable livelihoods approaches emphasizes three components: analysis of livelihood strategies and how they change over time, linkages between the micro and macro contexts of vulnerability [6], and access to capital assets [7]. The sustainable livelihoods approach has become part of the dominant paradigm among international development agencies and is the preferred approach according to PEPFAR guidelines for ES [2, 8]. The approach originated with the Brundtland Commission on Environment and Development in 1987 [9] and draws upon the scholarship of economist Amartya Sen, whose conception of “entitlements” laid the groundwork for asset-based analysis focused on livelihoods [5]. Assets are described according to the sociological concept of symbolic capital [10], with capital assets including human, natural, financial, social, and physical capital [11].

This study used a sustainable livelihoods framework to measure the concept of vulnerability as defined by the USAID/PEPFAR Health Office in Côte d’Ivoire, which is the degree of inability of households to provide for the health, education, and nutritional needs of HIV+ and HIV- household members to mitigate the economic and health impact of HIV, increase their ability to cope with infection, and reduce their risk for acquiring HIV. After conducting an extensive literature review of existing vulnerability assessment methodologies, we were unable to find an HIV-sensitive economic vulnerability assessment tool to meet USAID/PEPFAR’s needs precisely [12].

We sought to fill this gap in the literature and meet USAID/PEPFAR’s needs by: (1) developing a measure to describe the current state of vulnerability in the regions of interest to USAID/PEPFAR in Côte d’Ivoire, (2) examining whether vulnerability differs among households currently being served by USAID and its implementing partners (program “beneficiaries”) and nonbeneficiary households, and (3) developing a vulnerability measure that could be used in the future to target program beneficiaries and examine changes in vulnerability over time for households in communities defined as HIV-affected.

## Materials and Methods

Between March 7 and April 9 of 2015, we conducted a cross-sectional survey of households in 78 purposively selected villages or urban neighborhoods located within five health regions of Côte d’Ivoire: Gbôklê-Nawa-San Pedro (37 villages), Gbêkê (9 villages), N’zi-Ifou (15 villages), Indenié-Djuablin (9 villages), and Abidjan 2 (8 villages). The regions were selected by USAID/PEPFAR because they were defined as “HIV-affected,” meaning the population had a high prevalence of HIV and large numbers of orphans and vulnerable children (OVC), and villages were selected because they were located near service sites (clinical, OVC, and social centers) supported by PEPFAR.

We aimed to survey 375 beneficiary households and 375 nonbeneficiary households in each of the five regions for a total targeted sample size of 3,750 households. We determined that a sample of 375 households for each group of interest would provide 7 per cent precision for estimating vulnerability levels with a 95 per cent confidence interval. We assumed a base proportion of vulnerability of 50 per cent to be conservative. Calculations also assumed an intraclass correlation of 3 per cent to account for the multistage sampling design.

Data collectors were trained in research ethics, survey data collection, and the survey instrument, including briefing on classifying households using the PEPFAR categories. Data collectors visited selected households following the sampling plan and field procedures. After obtaining written informed consent, they conducted in-person interviews in French with self-identified household heads 18 years or older from the selected households, or if a household head was not available, another adult (18 years or older) who may be acting in place of the household head. Data were entered directly on mobile devices and uploaded daily to a secure server.

This study was reviewed and approved by the federally-registered institutional review board of FHI 360, the Protection of Human Subjects Committee, and the Comité National d’Éthique et de la Recherche (CNER) in Côte d’Ivoire.

### Instrument development

**Fig 2:**
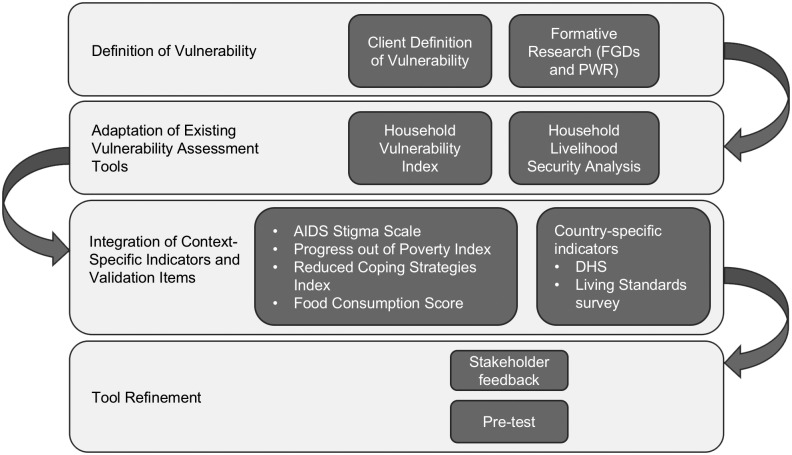
Instrument development process.

The survey instrument was developed in accordance with USAID/PEPFAR’s definition of vulnerability to generate vulnerability classifications based on PEPFAR’s ES categories (Fig 1). We followed a step by step process to develop the instrument (Fig 2), starting with formative qualitative research. The quantitative instrument was then developed based on two existing models—the Household Livelihood Security Analysis (HLSA) and the Household Vulnerability Index (HVI)—that were adapted to the Ivoirian context according to the definition of vulnerability indicated.

The formative qualitative study was conducted under a separate protocol to obtain locally-contextualized descriptions of vulnerability to inform indicators for the quantitative instrument and to test the feasibility of a participatory vulnerability ranking exercise as a method of identifying highly vulnerable households for program targeting. Thirteen focus group discussions (FGDs) were conducted with community members and stakeholders in four of the five study regions. Next, a pilot participatory vulnerability ranking (PVR) activity, adapted from Simanowitz and Nkuna’s 1998 manual for participatory wealth ranking [13], was conducted in three of the communities where the FGDs took place. In each location, FGDs were used to generate a local definition of vulnerability. Then, these definitions were used in separate FGDs for the PVR activity, where up to 30 participants ranked up to 50 households in their community in terms of relative vulnerability. The participants then classified ranked households as least vulnerable, less vulnerable, more vulnerable, and most vulnerable.

The formative research was used to adapt two existing sustainable livelihoods surveys to the local context. The HLSA framework is an assessment framework based on a sustainable livelihoods approach developed by CARE International [14]. This asset-based framework provided a starting point for assessing the dimensions of economic security, food security, health security, educational security, and empowerment in the Ivoirian context [15]. The HVI also uses a sustainable livelihoods approach, but it was designed to generate an index of the vulnerability of households and communities to the impacts HIV and livelihood shocks [16]. The HVI has a classification system similar to the PEPFAR ES categories (Fig 1) that identifies households that are vulnerable but coping as “coping,” households that urgently need assistance as “acute,” and households in dire need as “emergency.”

Our quantitative instrument was composed using the HVI survey templates developed by Development Data and the Food, Agriculture and Natural Resources Policy Analysis Network (FANRPAN) in 2008 [17] and 2013 [18] as a foundation. We also included indicators from an HLS survey conducted by CARE in Zimbabwe to contribute supplementary questions on health, livelihood sources, savings, expenses, and external sources of support [19]. Some of the general questions in these surveys were replaced by country-specific questions addressing the same indicators as found in Côte d’Ivoire’s latest Living Standards of Households survey [20] and the 2011–2012 Demographic and Health Survey (DHS) [21].

One dimension of vulnerability in USAID/PEPFAR’s definition is a household’s ability to provide for its members’ health needs. Although the HVI and HLS surveys used to inform our instrument include health indicators, additional country-specific health indicators were added from Côte d’Ivoire’s 2011–2012 DHS. Because the survey was meant to assess vulnerability experienced by populations in areas of high HIV prevalence, an AIDS stigma scale measuring negative attitudes toward people affected by HIV was added to the instrument. Stigma is related to vulnerability in that it can be a barrier to HIV prevention and treatment and can interfere with the livelihoods of those affected by HIV and AIDS. The nine-item AIDS-Related Stigma Scale was developed for the African context and validated in South Africa [22]. Because published documentation does not provide guidance on scoring the scale, we assigned one point to each of nine questions, where responses indicated the presence of stigma toward individuals affected by HIV. Scores range from zero to nine with higher scores indicating more stigma.

Several additional well-known scales were added to the instrument as validation items. These scales measure poverty and food security, which were expected to vary in similar ways to our measure on overall vulnerability. Based on a review of the literature on vulnerability, we anticipated correlations between poverty levels and vulnerability levels. Therefore, our instrument included the Progress out of Poverty Index (PPI), a 10-question scorecard developed by Mark Schreiner and the Grameen Foundation to rapidly assess the likelihood that a household falls under a given poverty line [23]. The PPI scorecard used was constructed using Côte d’Ivoire’s 2008 Household Living Standards Survey to assess the likelihood of whether a household’s expenditure placed it below the Côte d’Ivoire national poverty line (as of 2013) and the $2/day poverty line at 2005 International Purchasing Power Parity (PPP).

Another key dimension of our conception of vulnerability relates to food security and nutrition. To assess hunger levels and access to food, we added indicators from the Food Consumption Score (FCS) and Reduced Coping Strategies Index (rCSI). The FCS was developed by the World Food Program to gather information on food consumption frequency and dietary quality at the household level [24]. A generic scoring template was applied with FCS score ranges aligning into “poor,” “borderline,” and “acceptable” categories of household food security status. Lower scores on the FCS indicate less access to food and consumption of poor quality food at the household level. The Coping Strategies Index (CSI) was developed to appraise a household’s food security status based on the response behaviors, or coping strategies, they demonstrate [25]. A shorter version of the tool, the rCSI, was used for this study. It is considered as accurate as the full CSI, while reducing the total number of questions from twelve to five [25]. The rCSI yields a score measuring the frequency of various coping behaviors as weighted by their severity. A higher rCSI score indicates greater levels of food insecurity.

Data collectors were trained on the PEPFAR ES categories (Fig 1) to provide a subjective assessment of household vulnerability status at the end of each interview. Based on their observations, they classified each household as in destitution, struggling to make ends meet, prepared to grow, or not economically vulnerable. These classifications were used to assess the validity or our instrument because no other existing tool was found to meet USAID/PEPFAR’s broad definition of vulnerability.

Once our item pool was developed, a stakeholder advisory group, facilitated by the survey firm, also reviewed the items for local relevance during a daylong meeting in Abidjan. Twenty stakeholders representing 13 unique organizations working in Côte d’Ivoire included relevant staff from USAID/PEPFAR and also program implementers identified by USAID/PEPFAR. Lastly, we pretested the instrument by conducting 10 interviews with households in each of the five regions to refine the item pool and to test the format of items and response options with the intended study populations.

### Sampling design

The sample selection was stratified by health region and beneficiary status. To ensure the sample sizes for these strata, the sample selection for beneficiaries and nonbeneficiaries was conducted separately. For beneficiaries, the survey firm constructed a list of households served by the USAID/PEPFAR programs in each region using information obtained directly from the implementing partners in the regions. Next, a random sample of 375 households was selected from these lists in each region.

For nonbeneficiaries, a general household sample was selected, from which only a few would be expected to be beneficiaries. Households were selected in two stages. For the first stage, the primary sampling units (PSU) were the enumeration areas (EA) in the selected villages. The sampling frame of EAs was obtained by the survey firm from the National Statistics Institute of Côte d’Ivoire. In each region, we selected 25 PSUs with probabilities proportional to size using the estimated number of households provided in the sampling frame. For the second stage, the survey firm enumerated the eligible households in each selected PSU. Next, we selected 15 households randomly from these lists in each selected PSU.

Every effort was made to obtain the information on the selected households following the sampling plan described here. The survey firm made a minimum of three attempts to complete data collection at a selected household. The number of attempts made and the reasons why some households were missed were recorded in field logs. In each region and both samples, the lead statistician also randomly selected additional households as replacements. This was done only up to an additional 10 per cent of the sample and replacements were counted in the computation of the response rates.

Sampling weights were constructed to account for the actual number of households found during enumeration and to account for refusals and replacements.

### Data analysis

Our approach to assessing the level of vulnerability of each household in the sample was expected to reveal that a household’s location on a vulnerability scale involves multiple pathways. Thus, a pair of households sampled from the population that are equally vulnerable may not be vulnerable for the same reasons. Principal component analysis (PCA) was used to produce an economical description of these pathways with the goal of creating a short index or scorecard that could be easily deployed in the field to efficiently target vulnerable populations with a standard package of interventions to reduce their vulnerability. As a data reduction technique, PCA was used to inform a measurement strategy based on the identification of blocks of variables to be combined into a small number of composite scores reflecting different “components.” Variables were grouped into blocks in order to minimize the loss of information about vulnerability contributed by individual measures and provide interpretable composite scores to inform and evaluate policies that address vulnerability.

Variables included in the PCA were individually evaluated descriptively based on completeness and variability. Variables that were only applicable to a subsample of the target population were not submitted to the PCA. The number of components retained was determined based on an examination of a scree plot. The retained PCA solution was then rotated using an oblique rotation method (PROMAX). After rotation, components with three or more items with high loadings (> 0.4) were aggregated into a component. Items that loaded on multiple components or with low face validity were dropped. Next, mean scores representing each of the retained components were created from standardized items (mean = 0, std = 1) with high positive (≥ 0.4) or negative loadings (≤ -0.4) on a single component (reverse coding items, when necessary). Inter-item agreement for each mean score was assessed using Cronbach coefficient alpha. We combined components into an overall measure if they were highly correlated based on a visual examination of the correlation matrix estimated from the mean scores.

To assess the validity of the component scores from the PCA, we compared them to four other measures we expected *a priori* to be correlated. Agreement of the component scores to the PPI, FCS, and rCSI was assessed by correlation analysis using the measures as continuous scores. We also used ANOVA to compare the mean component scores that resulted from the PCA across the observed data collector classifications of the households into the PEPFAR ES categories.

Using the mean component scores, households were classified into four vulnerability levels using the locally determined distributions from the participatory vulnerability ranking activity described above. The average distribution (when rounded) found during that activity is as follows:

- Least vulnerable: 20%
- Less vulnerable: 30%
- More vulnerable: 45%
- Most vulnerable: 5%

We obtained cut points for the component scores to classify households in four vulnerability categories that reproduce the above distribution by calculating the appropriate percentile using the entire sample across regions and beneficiary status. We then compared the distribution across these categories when disaggregated by beneficiary status using Chi square tests for each region separately and for the regions combined.

Descriptive statistics include weighted means and percentages and unweighted frequencies to clearly indicate the sample size for each analysis. Statistical tests were performed using two-sided alternatives and a 5 per cent significance level. No imputation of missing values was performed except for the Food Consumption Score, where we assumed that households that responded to at least one but not all of the questions about particular food types did not consume those missing food types in the past seven days. Pair-wise deletion was used for the PCA.

## Results

A total of 3,754 households were interviewed. Due to a data transmission error, data are available from 3,749 households. The response rate was 97 per cent overall and ranged from 91 per cent of beneficiaries in Abidjan 2 to 100 per cent of nonbeneficiaries in N’zi-Ifou and Gbôklê-Nawa-San Pedro. Two households, one in N’zi-Ifou and one in Abidjan 2, were reclassified for the analysis from the nonbeneficiary sample to the beneficiary sample based on their self-report of receiving services from USAID/PEPFAR.

Households were largest in N’zi-Ifou, with a mean of six members (Table 1). Indenie-Djuablin had the smallest households, averaging five members. The average dependency ratio was highest in Gbêkê, at 77 per cent, compared to the lowest dependency ratio in Abidjan 2, at 60 per cent. 18 per cent of the surveyed households were headed by females. Education levels were highest in Abidjan 2 and lowest in Gbêkê, with 83 per cent and 58 per cent of household heads having attended primary or higher levels of education, respectively. Approximately 60 per cent of households had at least one member who had been bedridden at least three different times in the past year in all regions, except in Gbêkê where it was lower (39%).

**Table 1:** Select characteristics of households by region.^1^^,^ ^2^

Variable	Gbêkê (N = 749)	N’zi-Ifou (N = 749)	Indenié-Djuablin (N = 750)	Abidjan 2 (N = 748)	Gbôklê-Nawa-San Pedro (N = 753)	Total (N = 3749)
**Number of household members**						
Mean (StdErr)	5.08 (0.11)	6.37 (0.17)	4.83 (0.20)	4.99 (0.15)	5.36 (0.17)	5.12 (0.10)
Total	749	749	750	748	753	3749
**Age dependency ratio N (%)**						
Mean (StdErr)	77.47 (4.38)	69.41 (3.87)	64.24 (3.66)	59.58 (2.59)	75.43 (3.61)	64.90 (1.82)
Total	589	627	608	608	633	3065
**Female-headed N (%)**	264 (24.7)	210 (21.7)	242 (24.2)	250 (17.8)	192 (13.5)	1158 (18.3)
Total	728	745	729	742	728	3672
**Head attended primary or higher education N (%)**	423 (58.1)	454 (64.9)	457 (68.8)	571 (83.3)	480 (65.0)	2385 (76.0)
Total	723	745	721	732	724	3645
**Household member bedridden for at least 3 different times in past year N (%)**	364 (38.9)	493 (60.7)	423 (62.3)	462 (60.9)	479 (58.5)	2221 (58.2)
Total	749	748	747	744	752	3740
**Primary source of income N (%)**						
Salary	121 (18.1)	181 (26.5)	136 (22.9)	217 (42.8)	165 (22.7)	820 (35.1)
Informal labor	503 (73.3)	452 (61.9)	541 (68.2)	449 (51.0)	508 (71.7)	2453 (58.2)
External sources	65 (8.7)	97 (11.6)	48 (8.9)	63 (6.2)	44 (5.6)	317 (6.7)
Total	689	730	725	729	717	3590
**Most important source of savings N (%)**						
No savings	327 (52.4)	220 (28.4)	151 (19.2)	298 (34.2)	288 (34.0)	1284 (35.0)
Savings groups	15 (2.2)	10 (1.4)	35 (5.0)	63 (9.9)	26 (2.1)	149 (7.1)
Formal financial inst.	309 (41.1)	420 (59.9)	398 (59.9)	182 (36.6)	326 (49.8)	1635 (41.7)
Hiding	37 (4.2)	77 (9.7)	132 (15.3)	173 (18.3)	84 (13.8)	503 (15.5)
Other	0 (0.0)	4 (0.6)	2 (0.6)	3 (0.9)	6 (0.2)	15 (0.7)
Total	688	731	718	719	730	3586
**Incurred any unexpected household expenses in past 12 months N (%)**	298 (31.6)	357 (43.6)	247 (32.0)	456 (60.2)	124 (12.3)	1482 (46.7)
Total	748	749	750	748	748	3743
**Number of economic shocks experienced this year**						
Mean (StdErr)	1.10 (0.13)	1.69 (0.09)	1.30 (0.12)	1.92 (0.06)	0.87 (0.09)	1.61 (0.05)
Total	749	749	750	748	753	3749
**Number of correct ways (mentioned) a person can acquire HIV**						
Mean (StdErr)	1.79 (0.09)	2.10 (0.06)	1.88 (0.08)	2.51 (0.07)	2.55 (0.07)	2.39 (0.05)
Total	749	749	750	748	753	3749
**Number of correct ways (mentioned) a person can avoid getting HIV**						
Mean (StdErr)	2.12 (0.08)	2.18 (0.06)	1.98 (0.08)	2.39 (0.07)	2.27 (0.09)	2.31 (0.05)
Total	749	749	750	748	753	3749
**Households report they have adequate knowledge to cope with AIDS-related illnesses for family members N (%)**	430 (50.3)	289 (38.3)	531 (67.5)	515 (69.8)	568 (68.9)	2333 (65.9)
Total	725	748	740	724	722	3659
**Heads tested for HIV and received result of HIV test N (%)**	600 (64.5)	530 (62.3)	582 (62.7)	597 (69.1)	535 (45.4)	2844 (63.9)
Total	744	749	750	742	750	3735
**Poverty likelihoods (100% national poverty line)**						
Mean (StdErr)	21.99 (1.70)	26.10 (2.25)	18.30 (1.37)	6.94 (0.74)	27.27 (3.04)	13.63 (0.80)
Total	590	631	606	593	633	3053
**Poverty likelihoods (international PPP $2/day poverty line)**						
Mean (StdErr)	33.54 (2.03)	38.26 (2.63)	28.63 (1.68)	13.23 (1.10)	39.16 (3.50)	21.96 (1.03)
Total	590	631	606	593	633	3053
**Food consumption score (FCS) N (%)**						
Poor (0–21)	40 (9.3)	14 (1.9)	6 (1.1)	13 (1.4)	25 (3.4)	98 (2.6)
Borderline (21.5–35)	51 (7.0)	29 (3.5)	24 (4.9)	99 (8.5)	70 (8.5)	273 (8.0)
Acceptable (>35)	658 (83.8)	706 (94.5)	720 (94.0)	636 (90.0)	658 (88.1)	3378 (89.4)
Total	749	749	750	748	753	3749
**Reduced Coping Strategies Index (rCSI)**						
Mean (StdErr)	11.23 (0.69)	4.99 (0.67)	9.26 (1.00)	5.64 (0.62)	7.95 (0.95)	6.88 (0.42)
Total	376	351	327	327	350	1731
**Data collectors’ rating based on PEPFAR ES categories N (%)**						
Not economically vulnerable	123 (23.7)	65 (10.8)	8 (1.9)	37 (8.8)	57 (13.0)	290 (10.9)
Prepared to grow	88 (16.9)	216 (34.9)	92 (17.5)	146 (28.7)	167 (30.7)	709 (27.5)
Struggling to make ends meet	456 (50.6)	368 (46.6)	545 (70.2)	340 (42.9)	266 (37.7)	1975 (44.4)
In destitution	82 (8.8)	100 (7.7)	105 (10.4)	225 (19.5)	263 (18.6)	775 (17.2)
Total	749	749	750	748	753	3749

^1^ Weighted means and percentages and unweighted frequencies are presented.

^2^ Changes in the total sample sizes for each variable are a result of missing values.

N’zi-Ifou had the highest level of dependency on external sources of income compared to other regions, with 12 per cent of households citing external sources as their primary source of income. Households in Gbêkê were the most likely to report not having any savings, at 52 per cent, compared to the region with the lowest percentage, Indenie-Djuablin, where only 19 per cent of households did not have any savings.

The percentage of households that incurred any unexpected household expenses, such as a house repair or urgent medical treatment, in the previous 12 months varied across the regions from a high of 60 per cent of households in Abidjan 2 to a low of 12 per cent in Gbôklê-Nawa-San Pedro. The mean number of economic shocks the households experienced the year before the survey was 1.6 across all the regions.

Knowledge of HIV, measured by asking household members to list ways people can acquire and avoid getting HIV, did not vary across the regions. N’zi-Ifou households were least likely to report having enough knowledge to cope with AIDS (38%), whereas Abidjan 2 households were most likely at 70 per cent, closely followed by Gbokle-Nawa-San Pedro at 69 per cent. However, Gbokle-Nawa-San Pedro households were also least likely to possess a household head who had been tested for HIV and received the results, at 45 per cent compared to an average across all five regions of 64 per cent.

The poverty likelihoods of the study population were calculated according to selected poverty lines using the PPI. The average poverty likelihood for all regions at the national poverty line was 14 per cent and the likelihood of earning $2/day according to Purchasing Power Parity (PPP) calculations for 2005 was 22 per cent. Gbôklê-Nawa-San Pedro was the poorest region on both measures, with N’zi-Ifou second poorest, and Gbêkê the third poorest. By far, Abidjan 2 was the least poor.

Examining the Food Consumption Scores (FCS), we found that the majority (89%) of the study population fell under the “acceptable” range in food security status. At 84 per cent, Gbêkê had the lowest percentage in the “acceptable” range. Similarly, Gbêkê’s rCSI score indicated, by far, the highest level of food insecurity among the regions and more than double the most food secure region in the study, N’zi-Ifou.

Data collectors’ classification of each household according to the PEPFAR ES categories varied greatly across regions, with the percentage of households rated “not economically vulnerable” ranging from 2 per cent in Indenie-Djuablin to 24 per cent in Gbêkê, and the percentage of households rated as “in destitution” ranging from 8 per cent in N’zi-Ifou to 20 per cent in Abidjan 2. Data collectors classified approximately 11 per cent of the surveyed households as “not economically vulnerable,” 28 per cent of the households “prepared to grow,” 44 per cent as “struggling to make ends meet,” and 17 per cent of the households as “in destitution.” While the data collectors’ ratings were, for the most part, in line with other measures of vulnerability, some unexpected findings emerged. For example, despite having the lowest poverty levels and highest levels of education, Abidjan 2 households also had the highest percentage of households rated “in destitution” by the data collectors.

### Principal Component Analysis (PCA)

Out of 98 variables considered, 65 variables were entered in the PCA after evaluation of their suitability. Examination of the scree plot suggested four components be retained. The four components explained only 21 per cent of the total variance among the items; that is, 21 per cent of the spectrum of vulnerability present in the full set of variables considered. After rotation, 13 variables loaded on the first component, six on the second component, five on the third component, and four on the fourth component. Thirty-seven variables did not load on any of the first four components.

Before rotation, the first component explained 8 per cent of the total variance and is characterized by households’ ownership of assets or wealth, and food security. This suggests that one pathway to vulnerability in the study population is through the co-occurrence of these factors within the same household. The standardized Cronbach coefficient alpha for component 1 is 0.80, which suggests good reliability, or internal consistency, of the items in component 1. Coefficient alpha did not improve substantially by deleting any variables from the component.

Before rotation, the second component explained 5 per cent of the total variance and is characterized by households’ knowledge of HIV. The standardized Cronbach coefficient alpha for component 2 is 0.67, which suggests poor reliability of the items. Coefficient alpha did not improve by deleting any variables from the component.

Before rotation, the third component explained 4 per cent of the total variance and is characterized by households’ size and experience with shocks in the past year. The standardized Cronbach coefficient alpha for component 3 is 0.61, which suggests poor reliability of the items, and alpha did not improve by deleting any variables.

Before rotation, the fourth component explained 3 per cent of the total variance and is characterized by households’ receipt of remittances in the past year and building materials. This component comprises only categorical measures with little variability. The standardized Cronbach coefficient alpha for component 4 is 0.49, which suggests poor reliability of the items, and alpha did not improve substantially by deleting any variables.

Low correlation among the components indicated that we should not combine any components. In other words, the components appeared to be measuring different dimensions of vulnerability and should not be combined into one, unidimensional vulnerability measure.

### Description of components

Households were classified into four vulnerability levels using the locally determined distributions described above. Further examination of the distribution of the component across these vulnerability categories is used to describe component 1. The levels of internal consistency and correlation with the other indices (shown below) were not as high for the other three components and thus their distribution across vulnerability categories is not further discussed.

Thirteen items loaded on component 1 (Table 2). Vulnerable households were more likely to be female-headed, own fewer assets, and have fewer utilities/services in their homes. Vulnerable households were also more food insecure—they ate fewer meals the day before the survey, and when there was not enough food, vulnerable households were more likely to reduce the number of meals they ate, limit portion sizes, rely on less preferred/less expensive foods, or rely on help from a friend or relative. Vulnerable households were more likely to say they were “unsatisfied” or “very unsatisfied” with their life over the past 12 months and that their household was “worse off” compared with other households in their village.

**Table 2:** Component 1 household characteristics by vulnerability levels.^1^

Vulnerability Item	Least vulnerable	Less vulnerable	More vulnerable	Most vulnerable
	(N = 445)	(N = 874)	(N = 1974)	(N = 456)
**Female-headed household N (%)**	26 (8.0)	148 (12.2)	697 (23.6)	287 (51.0)
Total	441	859	1937	435
**Number of assets owned**				
Mean (StdErr)	11.77 (0.48)	7.91 (0.13)	6.40 (0.09)	3.80 (0.23)
Total	445	874	1974	456
**Number of utilities and services in home**				
Mean (StdErr)	4.36 (0.15)	3.26 (0.07)	2.76 (0.06)	1.88 (0.14)
Total	445	874	1974	455
**Ownership of media equipment N (%)**				
None	0 (0.0)	27 (2.6)	453 (18.3)	325 (73.8)
Only radio and/or television	72 (14.2)	477 (53.7)	1247 (63.3)	129 (22.4)
VCR/DVD and/or satellite dish	373 (85.8)	370 (43.7)	274 (18.5)	2 (3.8)
Total	445	874	1974	456
**Number of working fans owned N (%)**				
None	9 (0.9)	134 (12.0)	939 (39.6)	401 (76.7)
One	134 (34.1)	448 (51.3)	853 (50.1)	52 (22.7)
Two or more	301 (64.9)	292 (36.7)	181 (10.3)	3 (0.6)
Total	444	874	1973	456
**Number of working cell phones owned N (%)**				
None	0 (0.0)	2 (0.2)	39 (1.6)	81 (20.6)
One	3 (0.8)	45 (3.0)	380 (16.4)	189 (37.2)
Two or more	442 (99.2)	827 (96.9)	1555 (82.0)	185 (42.1)
Total	445	874	1974	455
**Number of meals eaten yesterday**				
Mean (StdErr)	2.97 (0.05)	2.62 (0.04)	2.22 (0.04)	1.82 (0.09)
Total	444	872	1970	455
**Number of times in past 7 days household…**				
Borrowed food, or relied on help from a friend or relative				
Mean (StdErr)	0.02 (0.01)	0.20 (0.03)	0.96 (0.08)	2.09 (0.19)
Total	444	867	1954	446
Reduce number of meals eaten in a day				
Mean (StdErr)	0.05 (0.02)	0.27 (0.03)	1.33 (0.07)	2.17 (0.19)
Total	441	863	1926	436
Rely on less preferred and less expensive foods				
Mean (StdErr)	0.13 (0.03)	0.99 (0.09)	2.22 (0.09)	3.47 (0.35)
Total	441	870	1952	446
Limit portion size at mealtimes				
Mean (StdErr)	0.04 (0.01)	0.49 (0.06)	1.58 (0.07)	2.66 (0.23)
Total	442	867	1942	438
**Feels “unsatisfied” or “very unsatisfied” with life over past 12 months N (%)**	41 (9.8)	232 (31.9)	1021 (59.7)	370 (84.6)
Total	444	873	1972	456
**Believes household is “worse off” compared with other households in the village or community N (%)**	7 (0.3)	50 (4.9)	445 (29.3)	275 (63.6)
Total	385	771	1776	431

^1^ Weighted means and percentages and unweighted frequencies are presented.

### Validity of the measurement model

To assess the validity of the component scores, we compared them to four other measures we expected *a priori* to be correlated (Table 3). We examined how the component scores compared across the data collectors’ assessments of the households according to the PEPFAR ES categories. The numbers are the mean component scores of the households in each category of vulnerability (as classified by the data collectors) with higher values indicating greater vulnerability. Given the component scores were computed by combining standardized measures for various numbers of items, the unit measure for the scores cannot be interpreted directly. These measures should be used to determine ranking and trends, but not to directly quantify changes in vulnerability status. The means for components 1, 2, and 4 are in the expected direction—the households that data collectors categorized as “in destitution” had the highest scores on these components, which indicates they were the most vulnerable, and the households categorized as “not vulnerable” had the lowest scores on these components. Moreover, the trends for all four categorizes were consistent with the mean scores for component 1, 2, and 4. While the mean component 3 scores were the lowest for the households categorized “not vulnerable,” as expected, no discernible pattern occurred among the other vulnerability categories. This suggests that component 3 does not correlate well to the data collectors’ assessments. The ANOVA tests for all components were significant, which indicates that at least one of the mean scores corresponding to a category is significantly different from the others.

**Table 3:** Comparison of component scores with data collectors’ assessment.

	Mean component scores				
PCA Component	Not economically vulnerable	Prepared to grow	Struggling to make ends meet	In destitution	p-Value
	(N = 290)	(N = 709)	(N = 1975)	(N = 775)	
Component 1	-0.65	-0.49	-0.12	0.31	<.0001
Component 2	-0.15	-0.01	0.08	0.24	0.0005
Component 3	-0.36	0.03	0.05	-0.08	<.0001
Component 4	-0.1	-0.04	0	0.13	0.0095

We examined correlations of component scores with the PPI, FCS, and rCSI (Table 4). Component 1 was moderately correlated (0.69) with rCSI and to a lesser degree with FCS (0.55) and PPI (0.46). Correlations of these measures with the other three components were low, which suggests that these components do not vary in the same way as the selected measures. The p-values shown in this table are testing whether a nonzero correlation exists. The (separate) correlations of components 2, 3, and 4 with the rCSI are not statistically significant, which indicates we found no evidence for an association between these measures. We also found no evidence for a significant correlation between component 3 and the FCS.

**Table 4:** Correlations of component scores with selected measures.

PCA Component	Selected Measure	Correlation	p-Value
Component 1	PPI	0.46	<.0001
	FCS	-0.55	<.0001
	rCSI	0.69	<.0001
Component 2	PPI	0.3	<.0001
	FCS	-0.17	0.0002
	rCSI	0	0.9418
Component 3	PPI	0.3	<.0001
	FCS	0.05	0.3009
	rCSI	-0.06	0.1723
Component 4	PPI	0.28	<.0001
	FCS	-0.09	0.0194
	rCSI	0.07	0.0890

### Vulnerability by PEPFAR beneficiary status

Across all five regions, beneficiaries and nonbeneficiaries differ on their component 1 (wealth/food security) mean scores (Table 5). Moreover, they differ in the expected direction with a larger proportion of beneficiary households having higher component 1 scores indicating greater vulnerability due to lack of wealth and food security. We did not detect a statistically significant difference in scores for component 2 (HIV knowledge), component 3 (household size/shocks), or component 4 (remittances/building materials) by beneficiary status.

**Table 5:** Vulnerability distribution of components by beneficiary status.^1^

	Nonbeneficiaries N (%)	Beneficiaries N (%)	Total N (%)	P-value^2^
	(N = 1865)	(N = 1884)	(N = 3749)	
**Component 1**				<.0001
Least vulnerable	319 (20.1)	126 (4.7)	445 (20.0)	
Less vulnerable	539 (30.2)	335 (15.4)	874 (30.0)	
More vulnerable	892 (44.8)	1082 (63.4)	1974 (44.9)	
Most vulnerable	115 (5.0)	341 (16.6)	456 (5.1)	
Total	1865	1884	3749	
**Component 2**				0.2251
Least vulnerable	243 (19.3)	486 (20.8)	729 (19.3)	
Less vulnerable	519 (28.0)	721 (34.7)	1240 (28.1)	
More vulnerable	1007 (47.6)	648 (43.4)	1655 (47.5)	
Most vulnerable	96 (5.1)	29 (1.2)	125 (5.0)	
Total	1865	1884	3749	
**Component 3**				0.1090
Least vulnerable	421 (19.9)	255 (10.8)	676 (19.8)	
Less vulnerable	599 (30.0)	570 (40.3)	1169 (30.1)	
More vulnerable	753 (44.9)	906 (42.5)	1659 (44.9)	
Most vulnerable	92 (5.2)	153 (6.5)	245 (5.2)	
Total	1865	1884	3749	
**Component 4**				0.8408
Least vulnerable	216 (13.3)	303 (15.4)	519 (13.3)	
Less vulnerable	251 (11.8)	216 (10.5)	467 (11.8)	
More vulnerable	1290 (69.7)	1260 (69.6)	2550 (69.7)	
Most vulnerable	108 (5.2)	105 (4.6)	213 (5.2)	
Total	1865	1884	3749	

^1^ Weighted means and percentages and unweighted frequencies are presented.

^2^ Rao-Scott Chi-Square test.

When the poverty likelihoods across all five regions (calculated according to two poverty lines using the PPI) are disaggregated by PEPFAR beneficiary status (Table 6), beneficiaries appear to be significantly poorer compared to nonbeneficiaries. Both poverty lines showed this trend.

**Table 6:** Other vulnerability measures by USAID/PEPFAR beneficiary status.^1^

	Nonbeneficiaries	Beneficiaries	Total
	(N = 1865)	(N = 1884)	(N = 3749)
**Poverty likelihoods according to 100% national poverty line**			
Mean (StdErr)	13.57 (0.81)	20.50 (3.72)	13.63 (0.80)
Total	1499	1554	3053
**Poverty likelihoods according to international PPP $2/day poverty line**			
Mean (StdErr)	21.87 (1.04)	30.98 (5.23)	21.96 (1.03)
Total	1499	1554	3053
**Food consumption score (FCS)**			
Poor N (%)	63 (2.6)	35 (1.6)	98 (2.6)
Borderline N (%)	116 (7.9)	157 (8.8)	273 (8.0)
Acceptable N (%)	1686 (89.4)	1692 (89.7)	3378 (89.4)
Mean (StdErr)	58.87 (1.06)	57.05 (3.49)	58.85 (1.05)
Total	1865	1884	3749
**Reduced Coping Strategies Index (rCSI)**			
Mean (StdErr)	6.84 (0.42)	12.02 (0.64)	6.88 (0.42)
Total	869	862	1731
**AIDS-Related Stigma Scale**			
Mean (StdErr)	1.71 (0.06)	2.36 (0.94)	1.72 (0.06)
Total	1606	1683	3289
**Data collectors’ classification of households N (%)**			
Not economically vulnerable	224 (10.9)	66 (5.2)	290 (10.9)
Prepared to grow	485 (27.7)	224 (8.4)	709 (27.5)
Struggling to make ends meet	917 (44.2)	1058 (62.6)	1975 (44.4)
In destitution	239 (17.2)	536 (23.8)	775 (17.2)
Total	1865	1884	3749

^1^ Weighted means and percentages and unweighted frequencies are presented.

Across all five regions the Food Consumption Score (FCS) does not appear to vary by beneficiary status. However, Reduced Coping Strategies Index (rCSI) scores were higher, which indicates greater food insecurity for beneficiaries compared to nonbeneficiaries.

Mean AIDS-Related Stigma Scale scores were higher for beneficiaries compared to nonbeneficiaries. However, these results are driven by one region (Abidjan 2), where we found a large disparity in stigma scores (higher stigma among beneficiaries) relative to other regions (data not shown).

When data collectors’ classification of each household according to the PEPFAR ES categories were compared by beneficiary status, a higher percentage of beneficiary households were classified into the two most vulnerable categories. A higher percentage of nonbeneficiary households were classified by the data collectors as “not economically vulnerable.”

## Discussion

### Overall Findings

Findings from the use of the composite measures, as well as the other indices measured in the study, suggest that USAID/PEPFAR serves the most vulnerable households in the study regions. The dimensions of poverty (measured by PPI), PEPFAR ES categories (based on data collectors’ ratings), AIDS stigma, and wealth/food security all showed this vulnerability focus. We did not detect a significant difference in vulnerability by beneficiary status on the dimensions of food security (measured by FCS), HIV knowledge, household size/shocks, or remittances/building materials.

Informed by previous research and theoretical frameworks of vulnerability, we approached our study by conceptualizing vulnerability as multidimensional. In an effort to determine the degree to which different vulnerabilities tended to occur together within the same household, we submitted 65 distinct measures of vulnerability to a principal component analysis (PCA). The goal of the analysis was to identify sets of correlated vulnerabilities and derive a small number of composite scores to create an index or scorecard that could be used to precisely and efficiently target vulnerable populations with a standard package of interventions to reduce their vulnerability. However, we found that the vulnerability measures did not cluster in ways that would allow for the creation of a small number of composite measures.

Although the measures did not coalesce into a single underlying dimension of vulnerability, the primary composite measure we developed on wealth and food security showed a good level of internal consistency. This measure also correlated with other indices measuring various dimensions of vulnerability. These findings suggest that a programmatic strategy addressing wealth and food security simultaneously may decrease vulnerability in some of the households in the study population. However, given the failure to capture a greater portion of the spectrum of vulnerability present in the full set of variables considered, this bundled approach is not likely to be effective in addressing the causes of vulnerability for the majority of households. The level of internal consistency and correlation with other indices was not as high for the other composite measures developed in our analysis. These findings suggest limited utility of the composite scores as comprehensive measures of vulnerability that could be used to examine changes in the population’s vulnerability over time. Instead, measures of changes over time should focus on specific aspects of vulnerability based on a narrower definition of the concept.

In our review of the literature of existing economic vulnerability assessments, we identified three studies that used similar methodology (that is, PCA) to develop vulnerability indices [26–28]. All three studies were focused on socioeconomic vulnerability related to environmental hazards, with variables focusing on assets related to rural livelihoods; environmental hazard exposure; and general socioeconomic characteristics, such as literacy, demographic characteristics, and access to health facilities. Because these studies had a much narrower conception of vulnerability in the context of rural livelihoods and environmental hazards than our study, they included a smaller and more homogenous set of variables in their analyses. This approach to variable selection may have resulted in composite scores reflecting more of the diversity in the full set of measures than our study did.

It is also possible that the variables related to productive assets included in our study were too general to apply to a heterogeneous population involved in diverse livelihood activities, and that including indicators of productive assets tailored according to livelihood type and disaggregated by geographical area, particularly across rural and urban settings, could have yielded stronger composite measures. For instance, assets that contribute to resilience against shocks in a rural household that depends on agriculture for its livelihood, such as livestock, may not be desirable or necessary in an urban household whose livelihood depends on informal vending, where other assets may be more effective. Detailed qualitative research can be used in future assessments to disaggregate key assets and shocks that factor into household vulnerability according to context.

Our instrument was based on a broad conception of economic vulnerability as related to HIV, for which an empirical definition has still not emerged from the literature, and which remains a complex, multifaceted subject of further study. Our results contribute to larger efforts to understand economic vulnerability and its relationship to HIV by demonstrating that a broad definition of vulnerability cannot be adequately captured by a simple index. Instead, these results suggest that numerous pathways to vulnerability exist in the study population and that households often have their own unique set of conditions that make them vulnerable. Despite demand for simplified targeting tools, our findings suggest that beneficiary households require individual assessment on multiple dimensions of vulnerability and a customized strategy is developed to address their specific needs. While a caseworker approach is more costly to implement than administering a scale to a sample of households, our data suggest this approach may be necessary to achieve a reduction in household economic vulnerability in the population studied.

### Limitations and strengths

USAID/PEPFAR’s definition of economic vulnerability suggests that household vulnerability has multiple HIV-related consequences, including the heightened risk of acquiring HIV infection, reduced ability to cope with infection, and reduced ability to mitigate the economic and health impact of HIV. Direct measures of these consequences would have served to validate our measurement model. The lack of such measures in our study means that we only have data to allow for a partial validation of our measurement model. Consequentially, the validity of our measurement model rests largely on the face validity of the various measures included in the PCA analyses, although we implemented an additional validation method by examining correlations of the components with other well-established indices. Measures of these HIV-related consequences, however, would have limited utility for the purposes of informing policy since they are (1) too expensive to collect on a scale that would permit routine monitoring of a large population, and (2) do not contain information on the factors that contribute to a particular household’s level of vulnerability.

We attempted to measure information from the five domains of capital assets according to a sustainable livelihoods framework: human, social, financial, natural, and physical capitals. However, based on stakeholder consultation, a limited number of natural capital variables were included in the survey. These variables included data on the total land available to a household and the proportion of land not used due to illness, death, or any other reason in the past season. Unfortunately, over 75 per cent of the households did not answer these questions (2,846 and 3,186 missing responses, respectively) so the two variables were discarded from the analyses. As such, natural capital is not represented in either the descriptive statistics or PCA in this study.

Some of the measures included in this study, such as the FCS, did not exhibit much variation in the study population. It is unknown if the lack of variability is due to poor sensitivity of the index or because the population is relatively food secure. It is also worth noting that though poverty likelihoods are based on the 2008 Living Standards Survey, which is the most recent national-level household survey data available, they are outdated and likely to underestimate actual poverty rates due to inflation.

Lastly, due to the cross-sectional design of our study, findings comparing vulnerability measures by beneficiary status should be interpreted carefully. It is not possible to determine if USAID/PEPFAR programming caused observed differences or if they are the result of selection. Longitudinal studies, preferably those that can incorporate randomization, are necessary to answer questions concerning causality.

Despite these limitations, our study has a number of strengths. The study benefited from a large, representative sample size and a high response rate, resulting in increased reliability of the estimates reported. The study also benefited from intensive formative research conducted in four of the five study regions; this research was used to inform the survey instrument and to identify a locally-defined vulnerability distribution, and the iterative approach we took to developing the instrument, including several rounds of local input. These steps increase the validity of our findings and ultimately their usefulness in guiding USAID/PEFPAR in its future programmatic planning efforts to reduce household economic vulnerability in Côte d’Ivoire.
